# Dr. Thomas W. Evans

**Published:** 1897-12

**Authors:** 


### Obituary.

### DR. THOMAS W. EVANS.

Dr. Thomas W. Evans died in Paris on November 15, 1897, of angina pectoris, after twenty-four hours' illness.

Dr. Evans recently visited the United States for the purpose of bringing the body of his wife to Philadelphia for interment. Upon the completion of this sad duty, he made an extended trip through the Middle, West, and Eastern States for the purpose, apparently, of making himself familiar with educational institutions, with the view of enabling him to carry out, intelligently, extensive benevolent projects he had in mind.

Dr. Evans was born in Philadelphia in 1823, in the portion then and now known as West Philadelphia, being west of the Schuylkill River. It is here, the most beautiful portion of the city, that he proposed, it is said, to found a museum of art. It is feared his sudden death may have prevented this and many other of his plans from being perfected, as it is supposed not sufficient time was given him to modify his will.

Dr. Evans received a common-school education in Philadelphia, and entered at an early age the gold-and-silversmith shop of Joseph Warner, on Merchant Street of that city, an old three-story brick house upon the ground now occupied by the Bourse. He and his brother both served the usual time of an apprentice to this calling, and became skilled in the manufacture of delicate silver and gold instruments intended for medical service. The wants of the dentists at that time, the present supply houses not being then known, induced Mr. Warner to undertake the business of supplying silver and gold solder for their use, and he occasionally added other work, but this latter was entirely exceptional, for the writer was quite familiar with the business at this and a later period. At this time, antedating the use of vulcanized rubber, medical instruments of a certain character were made of gold or silver, generally the latter gilded, and it required a marvellous degree of skill to solder some of these without injury.

This work brought the subject of this sketch in contact with dentists, and infused into his young and ambitious mind a desire to study this calling and make it the business of his life. He entered the office of Dr. J. D. White, then the most prominent dentist in Philadelphia, and subsequently he matriculated in Jefferson Medical College.

It frequently happens that the lives that men and women are to lead turn upon trifling incidents, and this was the case with young Evans. His skill as a mechanic enabled him to acquire the art of filling teeth at eighteen years of age superior to the majority of dentists then in practice. He exhibited his work, customary at that day, at the annual exhibition of mechanic arts under the care of the Franklin Institute, and secured a gold medal for his proficiency. The claim was then made that the fillings were inserted in the mouth and the teeth subsequently extracted.

This exhibit attracted the attention of Dr. Brewster, an American dentist of Paris, who eventually secured the services of Dr. Evans. This connection did not last long, for the ambitious young dentist opened his own office in Paris, and from this period the remarkable career, known the world over, began.

The statement made by the press that he was the first to introduce gold filling in Europe has no foundation in fact; that he improved methods then existing is possible and probable. It is very certain that it was due to his efforts that American dentistry abroad received its first impulse towards a character which it has maintained to the present time, through the superior work of many who have followed his footsteps as practitioners in European countries.

His skill attracted the attention of Napoleon III., and he became not only his professional adviser, but a friend. It was through this friendship for the Emperor that the latter formed the acquaintance with Eugénie, whom he subsequently made Empress of the French nation.

Dr. Evans was a born diplomat, and in that capacity performed essential service, in a quiet way, to Napoleon and to his native country. It is said that through his efforts an open rupture was prevented between the United States and France during the Civil War. His professional connections, and in many instances friendship, with the reigning houses of Europe gave him opportunities for secret diplomatic work, which Napoleon was not slow to accept. His practice may be said to have extended throughout Europe, for he was a frequent attendant, professionally, upon crowned heads and their families. From these he received almost innumerable evidences of their appreciation in the form of gifts and honors, which, in themselves, will make a valuable contribution to the museum which he had in contemplation to establish.

During the Crimean War he was sent by the Emperor, at his own suggestion, to study the sanitary condition of European camps and hospitals, and became so impressed with the misery and suffering there presented that he secured the interest of the civilized world in measures of reform. He was active in the Sanitary Commission during the Civil War, and it was largely due to his influence that the ambulance service during the Franco-Prussian War was placed on the excellent foundation it was at that time and has since continued.

His last service to the imperial family was the well-known assistance he gave the Empress in escaping from Paris. This is not necessary to detail here, but it was one of the most striking incidents of a life not devoid of many worthy of commemoration.

His wealth has been, probably, much overrated. While he had special opportunities for accumulation from practice, dentistry does not permit, even for the most favorably situated, the acquirement of millions. He had opportunities of knowing the changes which Napoleon proposed to make in the effort to beautify Paris and at the same time obliterate streets dangerous in time of revolution. With a keen sense of future possibilites he invested largely upon the lines of new avenues. In this way, mainly, were his large possessions secured.

It is difficult to reach conclusions as to Dr. Evans's standing as a dentist. That he was skilled in his profession there can be no question, but so are thousands of others. That he aided largely in raising the status of dentistry in Europe is certain; but it was through his personal qualities rather than from any active work in advancing its real interests. The only important service rendered, that the writer is aware of, was the active interest he took in the introduction of rubber as a base for artificial dentures. He published several books under his name, but beyond these he is almost unknown in dental literature or in dental scientific research. It is not given to every man to do this kind of work, and yet Dr. Evans performed a service that challenges recognition. He began his dentistry when, as he expressed it, "It was in the mire," and he left it an honored profession in all lands. His dignified character as a practitioner, combined with special advantages, aided in this work. In this direction he did more than the majority of dentists in his day and generation. This will, however, be his principal monument in dentistry, unless he has managed to arrange, prior to his death, for the great school of dentistry which seemed to occupy his mind when in this country.

The writer of this had the pleasure of meeting Dr. Evans a few days before he sailed for Europe. He was then full of life and energy, and the announcement of his death came with a shock usually accompanying all such sudden departures.

He leaves no children, but a large number of relatives in Philadelphia and elsewhere. Whatever may be the final distribution of his wealth, he will leave to dentistry the memory of a faithful exponent of its best principles and an example of devotion to its highest interests worthy of an enduring record in its archives.

